# The impact of frailty on the use of social services, medication and mortality risk: a cross-sectional study

**DOI:** 10.1186/s12877-024-05441-z

**Published:** 2024-10-23

**Authors:** Nanda Kleinenberg-Talsma, Fons van der Lucht, Harriët Jager-Wittenaar, Wim Krijnen, Evelyn Finnema

**Affiliations:** 1 Department of Science in Healthy Ageing and Healthcare (SHARE), University Medical Center Groningen, University of Groningen, Groningen, The Netherlands; 2 Research Group Healthy Ageing, Allied Health Care and Nursing, Hanze University of Applied Sciences, Groningen, The Netherlands; 3 FAITH research, Groningen/Leeuwarden, The Netherlands; 4 Centre for Health and Society, National Institute of Public Health and the Environment, Bilthoven, The Netherlands; 5 Aletta Jacobs School of Public Health, Groningen, The Netherlands; 6 Department of Gastroenterology and Hepatology, Dietetics, Radboud university medical center, Nijmegen, The Netherlands; 7 Faculty of Physical Education and Physiotherapy, Department Physiotherapy and Human Anatomy, Research Unit Experimental Anatomy, Vrije Universiteit Brussel, Brussels, Belgium; 8 Faculty of Science and Engineering, University of Groningen, Groningen, The Netherlands; 9 Department of Health Science, Section of Nursing Research, University Medical Center Groningen, University of Groningen, Groningen, The Netherlands; 10 Research Group Nursing Diagnostics, Hanze University of Applied Sciences, Groningen, The Netherlands; 11 Research Group Living, Wellbeing and Care for Older People, NHL Stenden University of Applied Sciences, Leeuwarden, The Netherlands

**Keywords:** Frailty, Older people, Social services, Community nursing services, Medication use, Mortality

## Abstract

**Background:**

Frailty is a common condition in older people, and its prevalence increases with age. With an ageing population, the adverse consequences of frailty cause an increasing appeal to the health care system. The impact of frailty on population level is often assessed using adverse health outcomes, such as mortality and medication use. Use of community nursing services and services offered through the Social Support Act are hardly used in assessing the impact of frailty. However, these services are important types of care use, especially in relation to ageing in place. In this cross-sectional study, we aimed to assess the impact of frailty on use of Social Support Act services, use of community nursing services, medication use, and mortality.

**Methods:**

We used a frailty index, the FI-HM37, that was based on data from the Dutch Public Health Monitor 2016, for which respondents ≥ 65 years of age were included (*n* = 233,498). The association between frailty, the use of Social Support Act services, community nursing services and medication use was assessed using the Zero Inflated Poisson (ZIP) regression method. Survival analysis using Cox proportional hazards regression was conducted to estimate the hazard ratios for the association between frailty and mortality.

**Results:**

The ZIP regression with a final sample size of 181,350 showed that frailty affected care use even after correcting for several covariates mentioned in the literature. For each unit increase in frailty index (FI) score, the relative probability of using zero Social Support services decreased with 7.7 (*p* < 0.001). The relative chance of zero community nursing services decreased with 4.0 (*p* < 0.001) for each unit increase in FI score. Furthermore, for each unit increase in FI score, the likelihood of zero medication use decreased with 2.9 (*p* < 0.001). Finally, for each unit increase in FI score, the mortality risk was 3.8 times higher (CI = 3.4–4.3; *p* < 0.001).

**Conclusions:**

We demonstrated that frailty negatively affects the use of Social Support Act services, the use of community nursing services, medication use, and mortality risk. This study is the first to demonstrate the impact of frailty on Social Support Act services and community nursing services in the Netherlands. Findings emphasize the importance of frailty prevention for older people and public health policy.

## Background

Frailty is a dynamic state, in which someone experiences deterioration in one or more health domains, increasing the risk of other adverse health outcomes, such as hospitalization, institutionalization, falls, or even death [1]. Frailty among older people is a growing issue. In recent years, the proportion of older people in the global population has increased, and as the risk of frailty increases with age, an increase in the number of frail older people is also becoming apparent [2, 3]. These same developments are visible in The Netherlands: by 2040, the number of Dutch adults of 65 years or older is expected to have risen to 30% of the population [4]. The adverse consequences of frailty not only negatively affect older people’s quality of life, but also health care use and costs [3]. Therefore, it is essential to understand the epidemiology of frailty and its impact on older people, policymakers, public health and the healthcare system.

Different adverse health outcomes are often assessed in relation to frailty, including mortality, to evaluate the impact of frailty. Multiple studies have shown that higher levels of frailty lead to untimely death [5–7]. Mortality is also an important and widely used criterion for determining the predictive validity of frailty instruments [8]. Moreover, the association between frailty and medication use has been demonstrated previously in terms of polypharmacy and the number of medications used [9, 10]. These studies showed an association between frailty and medication use.

However, other important types of care use have hardly been investigated in relation to frailty. The first is the use of community nursing services, which are nursing and care services at home, such as helping to get dressed or wound care. The second is the use of social services, which includes various services and facilities such as meal services, domestic help, or transport facilities. Social services are meant to assist with participation in society and the self-efficacy of citizens. Although studies in Ireland, Belgium and Australia demonstrated that higher levels of frailty were associated with higher levels of community health and social service use in these countries [11–13], similar recent studies about the Dutch context are lacking. Besides, some of these studies were based on physical frailty [11, 12], or aimed at specific target groups, e.g., men [12].

Since community nursing services and Social Support Act services are of great importance in relation to ageing in place, they are highly relevant to investigate in relation to frailty. In many Western societies, aging in place is becoming the predominant course for older people [14, 15]. In The Netherlands, for example, prevention and health promotion are arranged on a national level in several laws and acts, complemented by recent agreements related to health care and healthy and active living [16–18]. The predominant message in these acts and agreements is that people should live in their own homes for as long as possible and only rely on formal care when their own network of informal care is not sufficient anymore. When formal care is needed, public health officers, municipalities and community nursing organizations are important in organizing this. If higher frailty levels are indeed related to higher levels of care use and higher mortality rates, this has important implications for older people themselves, policymakers, the health care system, and public health. People with higher levels of frailty will experience a lower quality of life and face more challenges living independently [3], and in turn, will need to rely on health care services more often, which not only burdens the health care system but raises expenses as well [19].

Gaining insight into the impact of frailty on medication use, Social Support Act services, community nursing services, and mortality provides important knowledge for public health policies. It is especially informative for local public health officers, municipalities and community nursing organizations, as they play an important role in executing public health services and the recent agreements in The Netherlands related to health care and healthy and active living. Hence, in the current study, we aimed to investigate the impact of frailty on the use of Social Support Act services, the use of community nursing services, medication use, and mortality on a population level.

## Methods

A cross-sectional study was conducted to investigate the impact of frailty on the use of Social Support Act services, the use of community nursing services, medication use, and mortality. We used several health-related databases within the Remote Access environment of Statistics Netherlands (CBS) for the analyses, which are described in more detail below. Frailty was measured using a frailty index, the FI-HM37 [20]. This frailty index was constructed in a previous study, using health deficits from the Dutch Public Health Monitor. The FI-HM37 is a multidimensional instrument including the physical, social and psychological health domains, and suited for epidemiological purposes [21]. The current study builds on the previous results. Frailty levels were related to the use of Social Support Act services, the use of community nursing services, medication use, and mortality.

### Dutch healthcare system

The vision behind the healthcare system in The Netherlands is the availability of good quality health care for all Dutch inhabitants [22]. Practically, the healthcare system is arranged around four acts related to health. First, The Health Insurance act, introduced in 2006 and providing for (basic) medical care, medication and medical aids [22]. All Dutch citizens must have medical insurance, and private health insurance companies and healthcare providers are central in the implementation of the act. The use of community nursing services and medication as analyzed in this study, fall under the Health Insurance Act.Second, since 2015, the Long-Term Care Act, the Social Support Act and the Youth Act are providing for other types of care, and the implementation of these acts are largely under the responsibility of the Dutch municipalities [18, 22]. The use of Social Support Act services asinvestigated in the current study, fall under the Social Support Act.

### Databases

To assess the relationship between the outcomes of the FI-HM37 and the use of Social Support Act services, the use of community nursing services, medication use and mortality, five databases were used within the Remote Access environment of Statistics Netherlands (CBS): the Dutch Public Health Monitor; Social Support Act database; Community Nursing Services database; medication registrations; death registrations.

### Dutch Public Health Monitor 2016

The Dutch Public Health Monitor (DPHM) is a health-related survey conducted by the Community Health Services (in Dutch: GGD’en), Statistics Netherlands (CBS) and the National Institute for Public Health and the Environment (RIVM) [16]. For the Health Monitor 2016, information was gathered in all 25 regions of the Community Health Services. A letter of invitation was used to reach respondents, asking them to complete the survey online. A paper copy of the survey was included with the invitation letter in some Community Health Services regions, while in other regions, the paper survey was only provided in the case of non-response for online participation. Lastly, telephone or in-person interviews accounted for 0.1% of the survey data. In total 457,153 Dutch respondents from private households completed the survey, a response rate of around 40% .

The DPHM was used in a previous study [20], to develop a frailty index, the FI-HM37. For the development of the FI-HM37, only respondents of ≥ 65 years of age were selected (*n* = 233,498). The current study is based on this same set of respondents. More information about the development and realization of the DPHM can be found elsewhere [23].

### Social support act

To measure the Social Support Act Services’ use, we used the Central Administration Office (CAK) registrations from 2017 [24]. Registrations include information from all Dutch citizens aged ≥ 18 years, registered in the municipal personal records database (in Dutch: BRP), who used Social Support Act services. The database includes care that is financed by the Social Support Act (in Dutch: Wmo), and that is received as per appointment at the care provider or at the clients’ home.

### Community nursing services

This database contains information from declarations for community nursing services provided through the Health Care Act [25]. The use of these services is registered by Vektis, an organization that provides statistical information to the branch of care insurance, as well as individual care insurers [25]. The use of community nursing services in 2017 was assessed. The database includes registrations of citizens aged ≥ 18 years, registered in the municipal personal records database (in Dutch: BRP), who need care or nursing at home due to illnesses, conditions or limitations, and make use of community nursing services arranged via the Health Care Act.

### Medication use

Medication use was measured by the number of medicines used in 2017, as registered by the Care Institute. Included are medicines that are reimbursed by basis health insurance, including those provided at outpatient hospital pharmacies and to persons in residential homes for the elderly, excluding those provided in hospitals and nursing homes [26]. Types of medicine were identified in the database according to the WHO classification system of ATC (Anatomic, Therapeutic, Chemical) [26]. Medication codes that could not be appointed to an ATC code were excluded, e.g., codes specific to a pharmacy, or codes of other materials such as bandages [26].

### Mortality

Mortality rates for the respondents were obtained from a database of deaths registrations from the municipal personal records database (in Dutch: BRP). This database contains the date of death of all persons who deceased since 1 October 1994 up to February 2021, and were registered in the Personal Records Database (BRP) on the date of death. Additionally, the database contains the date of death of persons who are not residents but were once residents of the Netherlands and whose death date has been received in the Register of Non-Residents (in Dutch: RNI) [27].

### Connection between the databases

Information from the databases concerning care use and mortality, was added to the DPHM database using an anonymized, meaningless number to couple respondents throughout different CBS databases. The DPHM database was used as the basis: all respondents aged ≥ 65 years present in this database were traced in the other databases by means of the anonymized key and connected to the DPHM database. Hence, for all respondents of the DPHM database, we added information concerning the types of care use and mortality. In case of no use of the Social Support Act services, community nursing services, or medication, respondents received a score of zero.

### Outcome variables

#### Social support act use

Social Support Act Services vary and can entail domestic help, personal care, or facilities and appliances, such as a wheelchair or shower chair. For the current study, we summed the number of types of services used per person in 2017 to indicate the degree of Social Support Act Services. Hence, for each respondent we indicated how many different Social Support Act services one uses.

#### Use of community nursing services

For the current study, we used the yearly volume of community nursing services in 2017 per person, in hours. The volume is based on care that has been provided and the associated time unit. This indicates for each respondent the amount of time that one has been provided with care.

#### Medication use

For the current study, we were interested in the number of medication one receives. Therefore, the different types of medication someone uses were summed to a total score.

#### Mortality

To obtain mortality rates, the starting point of data collection for the DPHM, September 1, 2016, was subtracted from the date of death.

### Independent variable: frailty

Frailty was measured using a frailty index, the FI-HM37 [20]. This frailty index was constructed in a previous study, using health deficits from the Dutch Public Health Monitor. The FI-HM37 is a multidimensional instrument including the physical, social and psychological health domains, and suited for epidemiological purposes [21]. The current study builds on the previous results.

For the construction of the FI-HM37, a set of health deficits was selected in the physical, psychological and social health domains, in line with described procedures [28]. The deficits received scores between 0 and 1, where higher scores indicate a more negative outcome, e.g., more difficulties in walking, experiencing nervousness more often, or experiencing loneliness more often. The deficits were assessed and psychometrically investigated, resulting in an FI consisting of 37 psychometrically strong deficits: the FI-HM37. The overall frailty score was computed as a proportion, resulting in a number between zero and one: the summed deficit scores were divided by the total number of deficits in the scale, e.g. 8.50/37 = 0.23. An increase of 0.1 in the frailty score indicates an increase of 3.7 deficits. The closer to 1, the higher the level of frailty.

### Covariates

Several covariates were used in the analyses, to control for their effects on care use. We included the demographic variables age, gender, living with a partner, and ethnicity; the socio-economic indicators educational level and financial difficulties; the geographic indicator urbanization level; lifestyle factors smoking, alcohol use, body mass index (BMI), being compliant to exercise guidelines; and finally, we included variables related to participation/daily activities: doing voluntary work and giving informal care. All these variables were taken from the DPHM dataset.

Age was treated as a continuous variable. Gender (male/female), living with a partner (no/yes), and ethnicity (Dutch/non-Dutch) were analyzed as dichotomous variables. Educational level was operationalized in four categories: no or primary education; lower vocational or lower general secondary education; intermediate vocational or higher general secondary education; higher vocational or university education. The concept of financial difficulties was operationalized in four categories: no difficulties at all; no difficulties, but I have to watch my expenses; some difficulties; serious difficulties. The level of urbanization was operationalized in five categories, based on the address density in municipalities: not urbanized; hardly urbanized; moderately urbanized; strongly urbanized; extremely urbanized.

Smoking was operationalized in three categories (never smoked; smoked in the past, but not now; currently smokes). Alcohol use was operationalized in three categories as well (never used alcohol; used alcohol before, but not in the past year; currently uses alcohol). BMI was a continuous variable from low to high. Meeting exercise guidelines (does not meet standards; does meet standards) and doing voluntary work (no/yes) were operationalized as dichotomous variables. Finally, giving informal care was a continuous variable indicating informal care in hours.

### Analyses

To analyze the association between the amount of care use and the main variable of interest, i.e., frailty, the method of Zero-Inflated Poisson (ZIP) regression was used. This type of analysis can be used in the case of count data with both excess numbers of zeroes as well as counts [29, 30]. A ZIP model consists of a part for the counts, with Poisson regression coefficients for the predictive variables, and a zero inflation part for the number of zeros (no care) with a logit regression [29, 30]. The covariates used were the same for each of the types of care use: care use as predicted by FI-HM37, corrected for gender, age, living with a partner, ethnicity, education level, financial situation, level of urbanization, smoking, alcohol use, BMI, exercise guidelines, doing voluntary work, giving informal care. Respondents were the unit of analysis. Statistical analyses were conducted using R [31].

For analyzing mortality as an outcome, we conducted survival analysis by Cox proportional hazards regression to estimate the hazard ratios. The latter represents the change in mortality risk per unit increase in the level of frailty [32].

## Results

The mean frailty score for the study population according to the FI-HM37 was 0.19 (*SD =* 0.14). The final analysis sample consisted of those 181,350 respondents for whom all the data for the Frailty Index was complete. Hence, analyses were based on those respondents for whom a FI score could be calculated. Table 1 below presents descriptive information about the covariates used in the analyses. Table 2 presents descriptive information regarding the outcome variables, i.e., the different types of care use analyzed in the current study. The frequencies in this table indicate the large number of zeroes (no care use) in the care data.

**Table 1:** Descriptive statistics of the covariates used in this study. Descriptives are presented for the total sample as well as the final sample on which the analyses were based

Covariates	Total sample N*	Response %	Final sample N*	Response %
**Gender**	233,498	100	181,350	100
Male	112,026	48.0	92,078	50.8
Female	121,472	52.0	89,272	49.2
**Living with partner**	**230**,**397**	**100**	**180**,**098**	**100**
No	75,064	32.6	54,144	30.1
Yes	155,333	67.4	125,954	69.9
**Ethnicity**	**233**,**498**	**100**	**181**,**350**	**100**
Dutch	207,315	88.8	161,225	88.9
Non-Dutch	26,183	11.2	20,125	11.1
**Education**	**217**,**028**	**100**	**175**,**664**	**100**
No or primary education	24,704	11.4	17,007	9.7
Lower vocational or lower general secondary education	97,696	45.0	76,239	43.4
Intermediate vocational or higher general secondary education	48,098	22.2	40,915	23.3
Higher vocational or university education	46,530	21.4	41,503	23.6
**Financial difficulties**	**219**,**549**	**100**	**177**,**017**	**100**
No difficulties at all	116,971	53.3	96,172	54.3
No difficulties, but have to watch expenses	79,298	36.1	62,897	35.5
Yes, some difficulties	19,203	8.7	14,891	8.4
Yes, serious difficulties	4,077	1.9	3,057	1.7
**Level of urbanization**	**228**,**704**	**100**	**177**,**724**	**100**
Not urbanized	28,303	12.4	21,984	12.4
Hardly urbanized	65,454	28.6	50,669	28.5
Moderately urbanized	42,480	18.6	33,115	16.8
Strongly urbanized	61,903	27.1	48,437	27.3
Extremely urbanized	30,564	13.4	23,519	13.2
**Smoking**	**216**,**928**	**100**	**172**,**085**	**100**
Never smoked	75,583	34.8	57,821	33.6
Previously, but not now	115,828	53.4	94,176	54.7
Currently smoking	25,517	11.8	20,088	11.7
**Drinking alcohol**	**225**,**607**	**100**	**179**,**229**	**100**
Never drank alcohol	27,223	12.1	19,181	10.7
Previously, but not in the past year	17,980	8.0	13,557	7.6
Currently drinking alcohol	180,404	80.0	146,491	81.7
**Exercise guidelines**	**214**,**891**	**100**	**173**,**433**	**100**
Does not comply to standards	133,128	63.0	102,652	59.2
Does comply to standards	81,763	38.0	70,781	40.8
**Volunteering**	**221**,**258**	**100**	**178**,**520**	**100**
No	150,216	67.9	117,972	66.1
Yes	71,042	32.1	60,548	33.9
**Continuous variables**	**N**	**Mean (SD)**	**N**	**Mean (SD)**
Age	233,498	73.7 (6.7)	181,350	73.1 (6.4)
Giving informal care (hours)	216,924	2.4 (12.9)	178,520	2.45 (13.1)
BMI	219,607	26.3 (4.1)	175,437	26.3 (4.1)
**Independent variable**	**N**	**Mean (SD)**		
FI-HM37	181,350	0.19 (0.14)		

*Variations in N due to missing data

**Table 2:** Descriptives of the outcome measures under study. Descriptives are presented for the total sample as well as the final sample on which the analyses were based

Types of care use		Using care (*N*)*	Not using care (*N*)*	Mean (± SD)	Median	Max.	25th percentile	75th percentile
**Social Support Act Services**,** in number of services**	Total sample	22,586	210,915	0.13 (0.46)	0.00	11	0.00	0.00
	Final sample	14,430	166,920	0.11 (0.41)	0.00	11	0.00	0.00
**Community Nursing Services**,** in yearly volume in hours**	Total sample	27,697	205,804	0.97 (5.53)	0.00	454	0.00	0.00
	Final sample	16,745	164,607	0.82 (5.07)	0.00	372	0.00	0.00
**Medication use**,** in number of medications**	Total sample	213,621	19,880	6.01 (4.52)	5.00	35	3.00	9.00
	Final sample	165,220	16,130	5.80 (4.43)	5.00	35	2.00	8.00
		**Deceased N**	**Deceased%**	**Alive N**	**Alive %**			
**Mortality**	Total sample	28,650	12.3	204,808	87.7			
	Final sample	21,252	11.7	160,098	88.3			

*Using care indicates respondents making use of Social Support Act services, Community Nursing services, or using medication. Not using care indicates the number of respondents not making use of Social Support Act services, Community Nursing services, or not using medication.

The results of the ZIP regression analyses are presented in Table 3, and are reported below. The zero-inflation parts of the analysis are presented here, indicating the relative probability of having zero as an outcome, i.e., the relative probability of not using the type of care under analysis. Each outcome is contrasted to the unit increase in frailty score, i.e., the difference between an FI score of 0 and 1. Hence, a unit increase in FI score should be interpreted as the difference between having no deficits present at all, versus having all deficits fully present.

**Table 3:** Zero-inflation part of ZIP regressions for use of Social Support Act services, use of community nursing services and for use of medication, as predicted by the FI-HM37 and covariates¹

Zero-inflation model coefficients (binomial with logit link)	A: Use of Social Support Act services		B: Use of community nursing services		C: Use of medication	
	Model A1	Model A2	Model B1	Model B2	Model C1	Model C2
Parameter	Estimate (SE)	Estimate (SE)	Estimate (SE)	Estimate (SE)	Estimate (SE)	Estimate (SE)
Intercept	3.923 (0.042)***	14.899 (0.401)***	3.645 (0.017)***	11.074 (0.176)***	-1.735 (0.014)***	5.374 (0.184)***
FI-HM37	-10.554 (0.157)***	-7.612 (0.196)***	-5.746 (0.051)***	-4.035 (0.072)***	-3.939 (0.082)***	-2.926 (0.101)***
Gender		-0.386 (0.053)***		-0.003 (0.023)		-0.186 (0.021)***
Age		-0.153 (0.004)***		-0.107 (0.002)***		-0.059 (0.002)***
Living with partner		1.359 (0.054)***		0.410 (0.023)***		-0.303 (0.023)***
Ethnicity		-0.071 (0.073)		0.062 (0.034)		0.021 (0.031)
Education		0.260 (0.029)***		0.019 (0.011)		0.019 (0.011)
Finances		-0.440 (0.032)***		0.019 (0.015)		-0.073 (0.016)***
Urbanization		-0.038 (0.019)*		0.074 (0.008)***		-0.022 (0.008)**
Smoking		-0.179 (0.039)***		-0.148 (0.017)***		-0.109 (0.016)***
Alcohol use		0.328 (0.034)***		0.164 (0.015)***		0.016 (0.018)
BMI		-0.031 (0.005)***		-0.024 (0.002)***		-0.100 (0.003)***
Exercise		0.664 (0.071)***		0.480 (0.027)***		0.090 (0.020)***
Voluntary work		0.088 (0.067)		0.288 (0.028)***		-0.024 (0.021)
Informal care		-0.016 (0.002)***		-0.003(0.001)***		0.002(0.001)
Log-likelihood:	-5.136e + 04 on 4 Df	-3.16e + 04 on 30 Df	-1.764e + 05 on 4 Df	-1.203e + 05 on 30 Df	-5.334e + 05 on 4 Df	-4.088e + 05 on 30 Df

¹ Social support act is measured as the amount of services used; Community nursing services as the volume of services used in hours; Medication use as the number of prescribed medications. The final sample size was *N* = 181,350. Statistical significance is indicated by * = *p* < 0.05; ** = *p* < 0.01; *** = *p* < 0.001.

### Social support act services

The estimated ZIP regression model of Social support Act services without correcting for covariates (Table 3), indicated that the higher the level of frailty, the more likely one is to be making use of services through the Social support Act: for each unit increase in FI score, the relative probability of using zero social support services decreased with 10.55 (*p* < 0.001).

After correcting for covariates, the estimated ZIP regression model of Social Support Act services indicated that for each unit increase in FI score, the relative probability of zero Social Support services decreased with 7.6 (*p* < 0.001).

Besides the effect of our main indicator, frailty, on Social Support Act services, several covariates also affected the use of Social Support Act services. Specifically, the model showed that being female, being of older age, having more difficulties to manage financially, smoking currently, having a higher BMI and giving informal care (*p* < 0.001), and living in a more urban environment (*p* < 0.05), all decreased the relative probability of using zero Social Support Act services (*p* < 0.001). Hence, these circumstances seem to increase the use of social support. Living with a partner, having a higher education level, drinking alcohol and exercising, on the other hand, increased the relative probability of using zero Social Support Act Services (*p* < 0.001), implying that these circumstances decrease the use of social support.

### Community nursing services

The estimated ZIP egression model of community nursing services indicated that the higher the level of frailty, the more likely one is to be using community nursing services. More specifically, for each unit increase in FI score, the probability of zero services decreased with 5.7 (*p* < 0.001).

In the estimated ZIP regression model with correcting for covariates, the effect of frailty on the use of community nursing services remained: for each unit increase in FI score, the relative probability of using zero community nursing services decreased with 4.0 (*p* < 0.001).

Besides the effect of frailty on the use of community nursing services, the model showed that the covariates being of older age, currently smoking, having higher BMI, and giving informal care, were factors that decreased the relative probability of using zero community nursing services (*p* < 0.001). This implies that the described circumstances seem to increase the use of community nursing. Living with a partner, living in an urban environment, drinking alcohol, exercising more and doing voluntary work (*p* < 0.001) increased the relative probability of using zero community nursing services, or in other words, seemed to decrease the use of community nursing services.

### Medication use

The estimated ZIP regression model of Medication use, without correcting for covariates, showed that the higher someone’s frailty level, the more likely he or she is to be using medication. Results indicated that for each unit increase in FI score, the relative probability of zero decreased by 3.9 (*p* < 0.001).

In the estimated ZIP regression model with correcting for covariates, frailty still greatly affected medication use: the higher the frailty level, the lower the relative probability of zero medication use. More specifically, for each unit increase in FI score, the probability of using zero medication decreased with 2.9 (*p* < 0.001).

Furthermore, this model showed the effects of covariates on medication use: being female, being of older age, living with a partner, having more difficulties to manage financially, smoking currently, and having a higher BMI (*p* < 0.001), and living in a more urban environment (*p* < 0.05), all decreased the relative probability of using zero medication. Hence, these circumstances seemed to increase the use of medication. On the other hand, meeting exercise guidelines (*p* < 0.001), increased the relative probability of using zero medication, i.e., more exercise seemed to decrease medication use.

### Mortality

For the Cox regression analysis, a first model was estimated as a reference with the FI-HM37 as the only explanatory variable. In the second step, we included several covariates. The estimated Cox survival model in Model 1 of Table 4 showed that frailty increased mortality risk. For each increase in frailty level, the risk of death was 6.7 times as high (CI = 6.2–7.3; *p* < 0.001). In model 2, the covariates are added. The effect of frailty on mortality risk remained significant: an increase in frailty level indicated a mortality risk of 3.8 times higher (CI = 3.4–4.3; *p* < 0.001), adjusted for the covariates.

Regarding the effects of the covariates on mortality, being of older age and smoking indicated a higher risk of mortality (*p* < 0.001). On the other hand, being female, having a higher educational level, experiencing more financial difficulties, alcohol use, having a higher BMI, more exercise, doing voluntary work and giving informal care all appeared to decrease mortality risk (*p* < 0.01).

Living with a partner, ethnicity and level of urbanization did not influence mortality risk in older people.

**Table 4:** Coefficients, Hazard Ratios (HR as exp of coefficients), and CI for HR from Cox regression of mortality on several explanatory variables including the FI-HM37

Cox Regression analysis						
	Model 1			Model 2		
Parameter	Beta	HR	95% CI	Beta	HR	95% CI
FI-HM37	1.905	6.724***	6.189–7.305	1.344	3.834***	3.422-4.300
Gender				-0.223	0.800***	0.774-0827
Age				0.040	1.041***	1.038–1.043
Living with partner				-0.014	0.986	0.952–1.022
Ethnicity				-0.021	0.979	0.932–1.028
Education				-0.023	0.978**	0.961–0.995
Finances				-0.032	0.969**	0.947–0.991
Urbanization				0.003	1.002	0.990–1.015
Smoking				0.114	1.121***	1.093–1.150
Alcohol use				-0.044	0.957***	0.934–0.980
BMI				-0.008	0.992***	0.988–0.996
Exercise				-0.122	0.885***	0.855–0.916
Voluntary work				-0.091	0.913***	0.881–0.946
Informal care				-0.002	0.998**	0.997-1.000

The final sample size was *N* = 181,350. Statistical significance is indicated by * = *p* < 0.05; ** = *p* < 0.01; *** = *p* < 0.001.

## Discussion

The present study aimed to assess the impact of frailty on use of Social Support Act Services, community nursing services, medication use, and mortality. Frailty was assessed using the FI-HM37, a frailty index previously operationalized from the Dutch Public Health Monitor 2016 [20]. We have shown that higher levels of frailty are associated with more use of services through the Social Support Act, community nursing services, medication use, and a higher risk for mortality. Both the ZIP regression and the survival analysis revealed that even when controlling for covariates, the impact of frailty on care use and mortality risk still is present.

Our study results show that frailty uniquely explains health-related outcomes independent from covariates related to demographics (i.e., age, gender, living with a partner, ethnicity), related to socio-economic status (educational level and financial difficulties), related to geography (urbanization level), lifestyle (smoking, alcohol use, BMI, exercise), and related to participation/daily activities (voluntary work, informal care).

While many studies have been conducted concerning the impact of frailty on care use, these studies were in varying geographical contexts [11–13], were based on physical frailty [11, 12], or aimed at specific target groups, e.g. men [12]. Instead, our study specifically aimed to serve the broader public health domain. Firstly, by including social support Act services and community nursing services, which are largely arranged and provided for by public health stakeholders such as municipalities, community health services and social welfare organizations [18]. Secondly, using information from extensive, nationwide databases to measure impact of frailty aids the task of estimating care needs [33]. Specifically, concerning the public health task to care for older people [33], our findings can in turn contribute as a basis for developing collective preventive interventions and for promotion of healthy and active aging.

The current findings are in line with previous studies in Belgium, Ireland, and Australia, on the relationship between frailty and community nursing services and the use of Social Support Act services. These studies found that higher frailty levels indicated more use of community health services (e.g. physical therapist, dietician or general practitioner) and home care services (e.g. personal care or home nursing) [11–13]. Our findings also indicate that the more frail someone becomes, the more someone will use community nursing or Social Support Act services. In Dutch health care policy, ageing in place is stimulated, and the responsibilities to organize support and care for community-dwelling older people rest with the municipalities [14, 15]. Therefore, the association we found between frailty and care use is of great interest for local public health professionals as it underlines the need for preventive policy and intervention.

Furthermore, we found that the more frail someone becomes, the more medication someone uses. Although a negative association between frailty and medication use has been found several times in the literature, our findings differ in the direction of the association. While the current study found that higher levels of frailty are associated with higher levels of medication use, previous studies showed that medication use is associated with higher levels of frailty [34, 35], e.g., frailty was taken as outcome measure, and medication use was found to be predictive for frailty. These results emphasize the indisputable but complex relationship between frailty and medication use [9], and imply that medication use in older adults should be handled with caution, and carefully monitored. Particularly in light of the possibility that polypharmacy contributes significantly to the onset and progression of frailty in older persons and that lowering polypharmacy may be a tactic for either preventing or reducing frailty [9].

Finally, we found that frailty has a negative impact on mortality risk: a higher frailty level implies a higher risk of untimely death. This result confirms previous studies [5–8] in which different frailty measures were used (i.e. several frailty indexes and the Tilburg Frailty Indicator), different follow-up periods were analyzed (i.e. from 0 to 19 years) and different study populations were included (i.e. starting from 45, 55, 65 and 75 years of age). Our results suggest that the negative effect of frailty on mortality risk holds more general in a very extensive study setting.

Besides the impact of frailty on the use of Social Support Act services, community nursing services, medication use and mortality, several covariates were also found to affect care use. Previous research indicated that specific groups in the population, such as older people or women or ethnic minorities, are more prone to using health care [36, 37], and our study confirmed that persons who were older, of female gender, and with financial difficulties made more use of care services and medication. Furthermore, unhealthy lifestyle habits are associated with negative health outcomes in literature [38], and in our study results, this was reflected in more care use for respondents who smoke and who have a higher BMI. Also, older age and smoking were found to increase mortality risk. Since the results for factors like smoking and a high BMI could potentially be modifiable, they provide intriguing new directions for preventive public health policy.

Finally, the finding that providing informal care is associated with an increased relative probability of using Social Support Act services and community nursing services is remarkable. However, previous studies have indicated that being an informal caregiver negatively impacts self-rated health, mental health and physical health [39, 40]. The current study findings seem to reflect these negative impacts by increased care use. This finding is crucial in the light of the aging population and the expectation that even less informal caregivers will be available in the future [41]: it emphasizes the importance of support for informal caregivers and attention for their needs.

However, next to the negative associations of the covariates, we found several positive associations between the covariates and types of care use. Comparable with previous studies that show a positive effect of physical activity and an active (social) life on health outcomes [34], our study reflects those findings in lower care use for respondents who comply to exercise guidelines and are active in voluntary work. Besides, education level was found to decrease the use of social support and mortality risk, which is seemingly in line with previous research where frail respondents with lower educational levels were more prone to hospitalization [42]. The same is true for living with a partner, which decreases the use of social support services, community nursing services and mortality risk. A recent study found that support from one’s social network could positively impact medication use [43], and our results seem to extend this finding to other types of care use, as living with a partner could offer social support.

Strikingly, female gender, more financial difficulties, alcohol use, higher BMI, complying to exercise guidelines, doing voluntary work, and giving informal care, decrease mortality risk (*p* < 0.01). While some of these latter findings, such as lower mortality risk for those who exercise or do voluntary work, are plausible, others seem rather counter-intuitive. For example, the finding that alcohol use seems to decrease the risk of mortality, is not in line with previous research in which adherence to guidelines for alcohol use was associated with lower mortality risk [44]. However, according to a cohort study in China [45], alcohol use was not associated with increased mortality risk, and a longitudinal study even found a decreased risk of mortality for ‘moderate’ alcohol use [46]. Particularly these latter findings could imply that an explanation for our finding regarding alcohol use needs to be sought in the amount of alcohol that is consumed.

The findings regarding the impact of the covariates on care use are quite specific and cover a range of issues. Further investigation regarding the impact of these covariates on care use and mortality is recommended but falls outside the scope of the current paper.

The current study has several strengths worth mentioning. Firstly, the outcome variables use of Social Support Act Services, medication use, use of community nursing services, and mortality are all based on registrations by various institutions. Such registrations can provide objective data compared to self-reported care use data. Secondly, including Social Services and community nursing services as care use types provides information about the relationship between frailty and these less-studied types of care, which are important in the Dutch context. However, the current study has also limitations. Firstly, the information about using Social Support Act services, Community nursing services, and medication does not specify the type of services or medication used. For instance, it is impossible to pinpoint whether someone used the Social Support Act for appliances such as a shower chair or for more extensive personal care or transport facilities. It would be interesting to investigate whether frailty impacts different types of services differently. Secondly, the databases for the different types of care use might contain some uncertainty due to administrative errors or flaws in the registration process. Thirdly, inherent to the cross-sectional design of the study, it was not possible to make any predictions about the direction of the associations between the variables under study. Further research would be needed to establish causal associations.

The current study has implications for both practice and research. Firstly, on a policy level, the relationship between frailty and care use emphasizes the importance of preventive policies or interventions for frailty in older people. Since public health policies are aimed at prevention by promoting and protecting health in general, the results of this study could particularly guide municipalities in shaping their public health policies. Secondly, the impact of lifestyle-related and participation-related covariates on care use, which we found in the current study, is also highly important for policy. These indicators are potentially modifiable, providing an opportunity to influence them in a positive way by stimulating an active and healthy lifestyle. Furthermore, our study validates the FI-HM37 by confirming the association of frailty with adverse health outcomes. Hence, for future research this implies that the FIHM37 can be used for determining frailty levels in future editions of the Dutch Public Health Monitor to gain insights and trends in frailty levels in community-dwelling older people in The Netherlands over a more extended period.

## Conclusions

The present study confirmed the impact of frailty on negative health outcomes, demonstrated by an effect on medication use, community nursing services, Social Support Act services and mortality risk. This is a unique combination of health outcomes included in one study, and it implies that the more frail older people are, the more dependent they become on the healthcare system. Hence, the study provides a clear signal to all professionals in public health and health care to acknowledge the adverse consequences frailty can have and to anticipate this by developing preventive public health interventions.

## Data Availability

The results are based on analyses of non-public microdata from Statistics Netherlands by the authors. The data that support the study results are accessed through the Community Health Services, Statistics Netherlands and the National Institute for Public Health and the Environment, but restrictions apply to the availability of these data. Under certain conditions, microdata are accessible for statistical and scientific research. For further information: microdata@cbs.nl.
